# Recruitment strategies and retention rates for five National Dental PBRN studies

**DOI:** 10.1017/cts.2024.499

**Published:** 2024-03-22

**Authors:** Rahma Mungia, Ellen Funkhouser, David L. Cochran, Joana Cunha-Cruz, Valeria V. Gordan, Donald B. Rindal, Cyril Meyerowitz, Veerasathpurush Allareddy, Jeffrey L. Fellows, Gregg H. Gilbert, National Dental PBRN Collaborative Group

**Affiliations:** 1 Department of Periodontics, School of Dentistry, The University of Texas Health San Antonio, San Antonio, TX, USA; 2 Division of Preventive Medicine, School of Medicine, University of Alabama at Birmingham, Birmingham, AL, USA; 3 Department of Clinical and Community Sciences, School of Dentistry, University of Alabama at Birmingham, Birmingham, AL, USA; 4 Director of Practice-based Research and Associate Dean for Research, University of Florida, College of Dentistry, Gainesville, FL, USA; 5 Associate Dental Director for Research, HealthPartners Dental Group, HealthPartners Institute, Minneapolis, MN, USA; 6 Eastman Institute for Oral Health, University of Rochester School of Medicine and Dentistry, Rochester, NY, USA; 7 Brodie Craniofacial Endowed Chair, and Head of Department of Orthodontics, University of Illinois Chicago College of Dentistry, Chicago, IL, USA; 8 Kaiser Permanente Center for Health Research, Portland, OR, USA; 9 Department of Clinical & Community Sciences, Distinguished Professor and the James R. Rosen Chair of Dental Research Chair, School of Dentistry, University of Alabama at Birmingham, Birmingham, AL, USA

**Keywords:** Prospective studies, patient retention, practitioner retention methods, clinical study, dentistry, practitioner characteristics, practice characteristics, patient characteristics

## Abstract

**Background:**

We describe a retrospective assessment of practitioner and patient recruitment strategies, patient retention strategies, and rates for five clinical studies conducted in the National Dental Practice-Based Research Network between 2012 and 2019, and practitioner and patient characteristics associated with retention.

**Methods:**

Similar recruitment strategies were adopted in the studies. The characteristics of the practitioners and patients are described. The proportion of patients who either attended a follow-up (FU) assessment or completed an online assessment was calculated. For studies with multiple FU visits or questionnaire assessments, rates for completing each FU were calculated, as were the rates for completing any and for completing all FU assessments. The associations of practitioner and patient characteristics with all clinic FU visits, and with the completion of all assessments for a study were ascertained.

**Results:**

Overall, 591 practitioners and 12,159 patients were included. FU rates by patients for any assessment varied from 91% to 96.5%, and rates for participating in all assessments ranged from 68% to 87%. The mean total number of patients each practitioner recruited was 21 (*sd* = 15); the mean number per study was 13 (*sd* = 7). For practitioners, practice type and patient enrollment were associated with greater clinic retention, while only race was associated with their patients completing post-visit online assessments. For patients, age was associated with clinic retention, while female gender, age, race, and education were all associated with greater completion of post-visit online assessments.

**Conclusion:**

The Network efficiently recruited practitioners and patients and achieved high patient retention rates for the five studies.

## Introduction

Recruiting and retaining community-based clinicians and patient participants in clinical research are critical challenges that can cause long delays to a clinical study, shorten the study intervention’s duration, or require additional funds [1–5]. High retention is important in evaluating any longitudinal study. Poor retention leads to questions of validity. Even small differences in retention in the outcome of interest can bias the findings of a study [6–8]. Reviews of retention strategies [9–11] show that the more retention strategies employed, the better the retention. Incentives, both monetary and nonmonetary, improve retention the most consistently; reminder calls and letters also consistently improve retention, but to a lesser degree [9].

Practice-based research networks (PBRNs) accelerate science and support the translation of research into routine clinical practice [12]. However, PBRNs have constraints not faced in other research settings; specifically, studies can only require a modest amount of the patients’ and practitioners’ time and cannot unduly disrupt patient flow [13–15]. The National Dental PBRN Network (Network) has operated since 2005 and was integrated as a fully national Network in 2012; the Network is funded through the National Institutes of Health [16]. The goal of the Network has been to enroll a broad range of practitioners and practice types to optimize generalizability [14–16]. It has achieved this by developing a robust National infrastructure to support high rates of sustained participation among enrolled members [14–16].

The Network has adopted a broad range of approaches to recruit practitioners into the Network and its studies, including tailoring recruitment materials, advertising in Network and Association newsletters, postal and electronic mailing utilizing state licensing board member lists, snowballing (existing network members reach out to prospective members), conducting informational outreach events such as hosting informational webinars, seminars, symposiums, and exhibit booths at conferences, Quick Polls (short surveys) and contacting clinical and research leaders of societies, associations, and organizations [15]. Furthermore, the Network has also used various activities to engage practitioner members. These include providing continuing education credits for attending study training, webinars, seminars, symposiums, and annual meetings, engaging in presentations, publications, and Quick Polls [15]. These activities effectively sustained a high level of practitioner engagement in clinical research and its relevance to everyday clinical practice [15].

The structure of the Network has been described for the 2012–2019 period [17]. Briefly, the central administrative base is at the University of Alabama at Birmingham. The Coordinating Center (CC), based at Westat in Rockville, MD, provided expertise in study design and statistical support and developed and maintained databases. The network comprises six geographic regions, each with a regional director and coordinator (RC) for administrative purposes. The RCs provide context about office flow, operations, and conducting a study in a busy dental practice venue. They coordinate recruitment by gauging practitioner interest in the study. They also help standardize activities (training, data collection, practice monitoring, and study closeout) and communication across regions. Most importantly, they recruit and train practitioners on study procedures and monitor the procedures performed by practitioners and office staff. They also perform quality management activities, monitor practitioners and office staff, study procedure execution, and respond to data queries.

This report describes the following for the five national prospective clinical studies conducted in the Network between 2012 and 2019: (1) practitioner and patient recruitment strategies; (2) patient retention rates; (3) strategies used to achieve these patient retention rates; and (4) practitioner and patient characteristics associated with patient retention [18–23]. The 2012–2019 period was chosen because it was the first fully national funding cycle, and it represents a time period when the same recruitment and retention strategies were used for all studies. By providing examples of proven effective methods in the Network, the over-arching goal of this report is to inform the clinical research community regarding planning and maximizing recruitment and retention of community-based practices for clinical research.

## Methods

### Practitioner recruitment and retention strategies

Participation in the Network and its studies is a phased process. Initial recruitment into the Network happens when dentists (and practice personnel) enroll in the Network by completing a Network Enrollment Questionnaire (EQ), which is required for network membership. The EQ queries practitioner characteristics such as the practitioner’s gender, race, year graduated from dental school, type of practice, specialty training, type and frequency of dental procedures performed, and administrative/geographic region of practice. The Network tracks EQ enrollment nationally/regionally through a database. The RC coordinates and monitors all study-related activities within the region, including research-ready (tasks that need to be completed to participate in any clinical Network study) and study-ready tasks (tasks that need to be completed to participate in a specific clinical study) (Table 1). The database allows RCs to record research study readiness tasks and use the continuously updated Network practitioner database that organizes and verifies the most up-to-date personal and practice information of Network members to help identify members for a clinical study.

**Table 1.** Tasks required by the practitioner, and some staff, to complete before participating in any study and then for specific studies

Tasks	Complete Online or in-person	Time to complete (minutes/days)	Administrative signatures required
Research Readiness tasks, those required before participating in any clinical research study. *(These can vary by region)*			
Enrollment questionnaire	online	15 minutes	No
Human subjects training	online	*vary by region*	Yes
Individual Investigator Agreement (IIA)*	online	5–10 minutes	Yes
Study readiness tasks, those required for each specific clinical research study.*(These do not vary by region)*			
Practitioner Institutional Review Board (IRB) approval	online	*vary by region*	Yes
Master Service Agreement (MSA)**	online	5–10 minutes	Yes
Study Protocol training (varies by study)	online or in-person	45–90 minutes	Yes

* IIA- A mechanism by which a practitioner can affiliate with an IRB.

** MSA- A contract in which the parties agree to a statement of work, payment requirements, work performance regulations, liability, and related matters.

Similar Network recruitment strategies were adopted for recruiting practitioners/patient participants for the five clinical studies. Live events, such as professional meetings, conference booths, or the Network regional annual practitioner meetings, were used. As a result, RCs have the opportunity to discuss clinical studies in detail with potential candidates face-to-face. Moreover, results of past/current studies are presented and discussed in break-out sessions at annual practitioner meetings, which engages members and creates enthusiasm to participate in upcoming clinical studies. Furthermore, webinars and symposium events cultivated practitioner snowballing, where existing members reach out to prospective members.

Telephone calls (typically by RCs, whom the office staff and practitioner know) to dental practices were used to speak directly with practitioners and encourage them to participate in a study of potential interest. Based on conversations with practitioners and RCs, calling between 8 am and 9 am was the most responsive time to reach practitioners, successfully increasing interactions with the practitioner and practitioner recruitment into a study. The postal mailing was adopted widely to recruit new members and invite them to participate in ongoing studies. Emails have effectively explained ongoing studies and provided pertinent information without disrupting practitioners’ daily lives. Additionally, a weekly or biweekly follow-up email was sent to practitioners, including updates on clinical study enrollment status. Monthly newsletters were also emailed, including information about the clinical study and practitioners’ testimonials to entice practitioners to join the clinical studies.

Upon practitioners’ expression of interest in a study, RCs email instructions on completing the study’s prerequisites (Table 1), including the human subjects training. Once the requirements were complete, practitioners were trained on study procedure implementation, including enrollment and consenting eligible patients. The Network retention strategies and tactics were designed to keep practitioner and patient participants from discontinuing the clinical study. The RCs maintain strong, positive relationships with the practitioners and have intermittent contact as necessary throughout the study to ensure their continued engagement. A continuously updated Network practitioner database allowed investigators and RCs to communicate effectively with practitioners, collect data, and receive datasets for all clinical study protocols. In addition, study training manuals are developed, and coordinators are trained to anticipate problems early to enhance practitioner adherence to the study’s protocols and prevent them from withdrawing from ongoing research.

### Patient recruitment and retention strategies

In optimizing patient recruitment for National Dental PBRN studies, dental practitioners employ a range of diverse strategies. Patient recruitment flyers strategically placed within the dental office serve as informative tools, succinctly outlining study details and actively encouraging patient participation. These visually engaging materials not only raise awareness about ongoing research but also provide patients with a tangible and accessible source of information during their dental visits.

Concurrently, dental practitioners conduct targeted patient chart reviews on scheduled appointment days, employing a systematic approach to identify potential participants who meet predefined eligibility criteria. This real-time assessment streamlines recruitment by pinpointing individuals most likely to qualify, enhancing efficiency, and ensuring a more focused effort. To bolster these initiatives, practitioners leverage their relationships with patients during routine appointments, discussing the study’s significance and benefits in a personalized manner that fosters trust and understanding, motivating patients to engage in the research process actively.

Furthermore, dental practitioners introduce compelling incentives for study participation, conveying remuneration options as a tangible acknowledgment of patients’ contributions and enhancing the attractiveness of involvement. The active engagement of dental office staff is usually pivotal in this comprehensive strategy, as staff are equipped with study information and training to ensure a unified and informed promotional effort within the practice. This collaborative approach establishes a supportive environment that consistently encourages patient involvement in National Dental PBRN research studies, ultimately optimizing patient recruitment and ensuring the success and impact of dental studies.

Practitioners or their staff instruct patients when enrolling on the importance of completing the study to retain them for the study’s duration. For in-person follow-up (FU) visits, confirming contact information (the patient’s and/or one contact person’s) is essential. Similarly, confirming the email address and telephone number minimizes the loss of patients for online FU assessments. Practitioners’ staff first attempted the process of contacting patients for FU visits. If unsuccessful, the RC and CC initiated tracking procedures to identify updated patient contact information and contact them. For some studies, the CC assisted with FU reminder calls.

### Study design

A retrospective analysis of prospective national clinical studies conducted by the Network during the 2012–2019 funding period was conducted. The Network conducted eight such studies during 2012–2019. To be included in the analysis below, each study had to be national in scope and had to require all enrolled patients to either come in for an FU visit or complete a post-visit online assessment. The CC maintained the database for each study conducted by the Network and the practitioner database (which includes the EQ). These data and accompanying documentation, including study protocols, data forms, and data dictionaries, are stored on secured servers. All clinical studies (viz., studies that enrolled practitioners and their patients) queried patients on demographics, including their sex, age, race, ethnicity, education, and dental insurance. Each study database had a practitioner identification number to allow merging with the EQ so that practitioner characteristics (e.g., demographics, practice setting, training) could be included with the study data (and were for the analysis below). For clinical studies with FU assessment(s), there was an indicator variable for attending or completing each requested FU assessment and the date of the assessment. For studies with multiple FU assessments (whether in person or online), two outcome variables were defined: whether completed any and whether completed all assessments. These were the primary outcome measures of retention.

The eight clinical studies conducted by the Network are presented in Table 2. The Network successfully enlisted the desired number of practitioners (150–200) and patients (1,700–3,800) for each study (Table 3). The studies proposed target recruitment numbers slightly beyond the number necessary to detect the desired magnitude of effect as statistically significant, while also ensuring compliance with IRB regulations by avoiding over-enrollment. Enrolling more than the target number requires submitting protocol deviations to the IRB. When recruiting/enrolling on a national scale, it can be challenging to stop enrollment at a specific date, especially when simultaneously wanting to maintain optimal relationships with practitioners who have devoted significant effort to a specific study. Three studies were excluded: (1) Suspicious Occlusal Caries study because patients were not followed [24]; (2) Anterior Open bite study because only patients whose treatment was considered ended or completed, viz., open-bite closed, were requested to return in 18 months [25]; and (3) Risk for Oral Cancer study because only patients with a positive high-risk human papillomavirus test (*N* = 11) result were requested to come in for a 6-month visit [26].

**Table 2.** Prospective clinical studies conducted by the National Dental Practice-based Research Network

	Enrollment			Follow-up		
Study name	# practitioners	# patients enrolled	Calendar time	Timing	*N*	%
**Included In Analysis**						
**In person/clinic follow-up visits**						
Cracked Tooth Registry (CTR)	209	2,858	04/2014–04/2015	Year-1	2,507	87.7%
				Year-2	2,236	78.2%
				Year-3	2,079	72.7%
				Any	2,617	91.6%
				All	1,947	68.1%
Factors for successful crowns (CROWNS)	205	3,828	03/2016–12/2016	21–42 days	3,749	97.9%
**Online follow-up assessments**						
Management of dentin hypersensitivity (MDH)	171	1,868	04/2015–12/2015	Week-1	1,645	88.1%
				Week-4	1,701	91.1%
				Week-8	1,696	90.8%
				Any	1,802	96.5%
				All	1,539	82.4%
Management of painful temporomandibular disorders (TMD)	185	1,886	10/2016–07/2018	1-month	1,770	93.8%
				3-month	1,740	92.3%
				6-month	1,716	91.0%
				Any	1,820	96.5%
				All	1,647	87.3%
Predicting outcomes of root canal treatment (PREDICT)	153	1,719	04/2017–11/2017	1-week	1,564	90.1%
				6-month	1,436	83.5%
				12-month	1,421	82.7%
				Any	1,569	91.3%
				All	1,351	78.6%
**Excluded From Analysis^1^**						
Suspicious Occlusal Caries (SOCL)	93	1,593	04/2015–09/2015			
Anterior Open Bite (AOB)	91	347	10/2015–2/2017			
Risk for Oral Cancer (ROC) study	37	1,025	08/2016–2/2017			

1 Reason excluded: SOCL–No patient follow-up required; AOB–Only patients whose treatment was considered complete were requested to return in 18 months (N = 254 did); ROC–requested to come in for a 6-month visit (N = 11).

**Table 3.** Target numbers

	# practitioners		Patients	
Study name	Target	Enrolled	Target	Enrolled
Cracked Tooth Registry (CTR)	150- 300	209	3,000	2,858
Factors for successful crowns (CROWNS)	200	205	4,000	3,828
Management of dentin hypersensitivity (MDH)	180	171	2,520	1,868
Management of painful temporomandibular disorders (TMD)	200	185	1,980	1,886
Predicting outcomes of root canal treatment (PREDICT)	175	153	2,000	1,719

Two out of the five studies described below required in-person FU visits. The Cracked Tooth Registry (CTR) study was a prospective, observational 3-year cohort study of posterior teeth with visible cracks. The purpose of CTR was to ascertain the rate of crack progression and identify characteristics associated with this progression [18,19]. Patients were requested to return for 3 annual recall visits. The Factors for Successful Crowns Clinical Study (CROWNS) was the only other study that required an in-person FU visit, and this visit was for the insertion/placement of the crown within 40 days [22].

The remaining three studies only requested patients to complete FU assessments electronically, namely, online or via telephone. The Management of Dentine Hypersensitivity (MDH) study, to understand the multiple treatments used for dentin hypersensitivity, requested 3 FU assessments (at 1, 4, and 8 weeks) to be completed [20]. The Management of Painful Temporomandibular Disorders (TMD) study identified factors contributing to TMD treatment decisions and requested FU assessments at 1, 3, and 6 months [23]. Finally, the Predicting Outcomes for Root Canal Treatments (PREDICT) study investigated risk factors for severe pain following a root canal and requested FU assessments 1 week, 6 months, and 12 months after completion of root canal therapy [21]. The number of practitioners, patients, calendar time involved, and the type and timing of FU assessments are presented in Table 2.

### Statistical methods

Data from the five studies were merged to create a practitioner-level and a patient-level dataset. Composite measures of follow-up were defined at the patient level (studies included in the measure are in parentheses): completed any follow-up (clinic or online: all studies included), completed any clinic follow-up (CTR and CROWNS), completed any web follow-up (MDH, TMD, PREDICT), completed all clinic follow-up visits (CTR only), completed all online follow-up for at least one study (MDH, TMD, PREDICT). Because the CROWNS follow-up visit was “insertion” of the crown (a procedure necessary to complete the treatment that was started at the study’s enrollment and which the patient would need to do regardless of study participation), this visit was not included in the measure of completing all clinic follow-up visits. Separate analyses were performed for clinic recall visits and for post-visit online/telephone assessments as associated practitioner and patient characteristics may differ for the two types of follow-ups.

Because some practitioners participated in multiple studies and would have different ages, their age during recruitment of the first clinical study (CTR) of this funding cycle, viz., 2014, was used in analyses described below. Descriptive statistics (proportions, means, standard deviations [sd], medians, inter-quartile [IQR], and overall range) of the practitioner and patient characteristics, primarily demographic, are presented. The associations of practitioner and patient characteristics with each of the composite measures of follow-up described above were ascertained. The significance, after adjusting for the clustering of patients within the practice using generalized estimating equations implemented with PROC GENMOD in SAS, was determined for each practitioner and patient characteristic separately. Each variable was then entered into a (full) model. Analyses of each study separately are presented in supplemental tables 1-5. All analyses were performed using SAS software (SAS v9.4, SAS Institute Inc., Cary, NC).

Furthermore, the study conformed to recognized standards of the US Federal Policy for the Protection of Human Subjects by seeking Institutional Review Board (IRB) approval for the five studies as described in study publications [18–23].

## Results

### Practitioner characteristics

**Figure 1.**
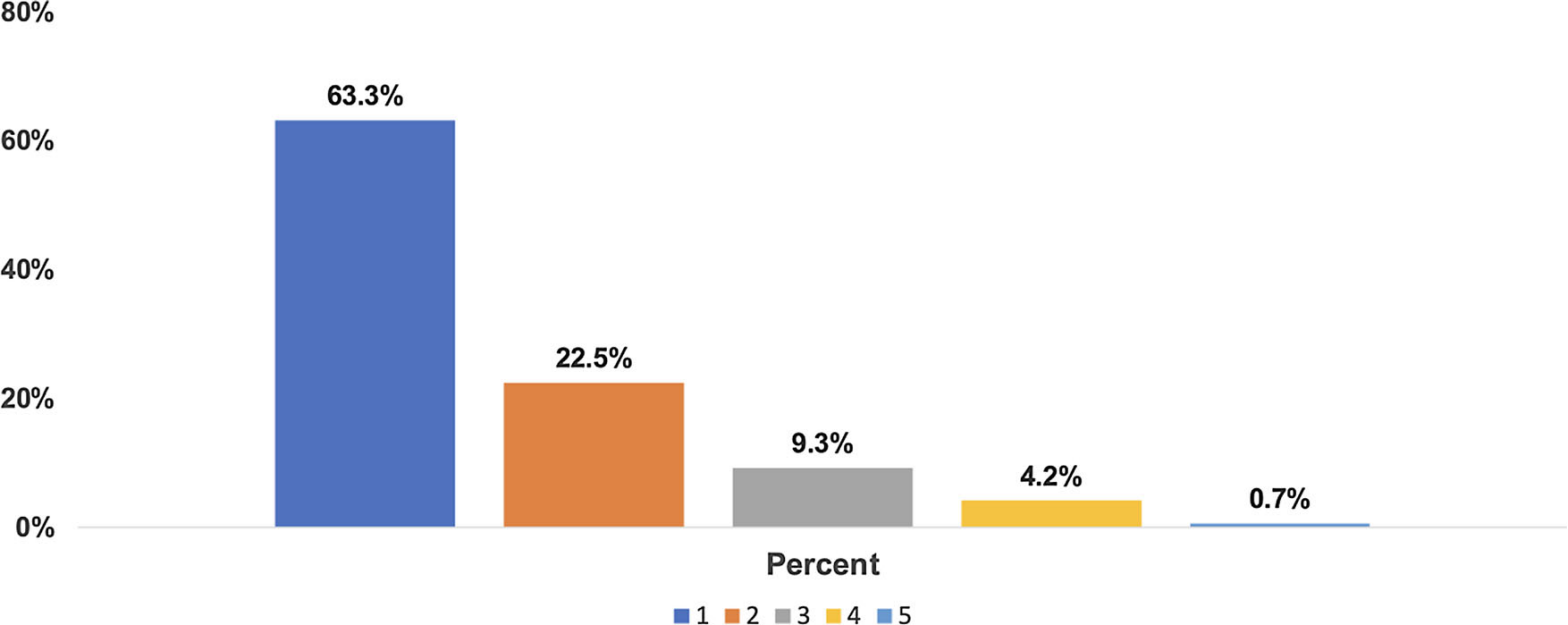
Percentage of studies practitioners participated in.

Overall, 591 practitioners enrolled patients in one or more of the five clinical studies: 374 (63%) in one study, 133 (22%) in two studies, and 84 (14%) in three or more studies (Fig. 1). Their mean age in 2014 was 50 (*sd* = 11). Most of the practitioners were male (70%), non-Hispanic White (78%), worked in private practice (84%), and were general practitioners (85%) (Table 4). Specialists only comprised 1 to 6% of practitioners for three of the studies (CTR, CROWNS, MDH). In contrast, specialists comprised 19% and 29% of practitioners in the TMD and PREDICT studies, respectively, which was expected, given the clinical conditions being investigated (orofacial pain and endodontics). Findings from these analyses are provided in the supplemental tables 1-5. All regions were represented (13% to 22% from each region). The mean number of patients a practitioner enrolled was 21 (*sd* = 15); the mean number enrolled per study was 13 (*sd* = 2). The number of patients enrolled by a practitioner varied across the studies, e.g., in the MDH study, enrollment was capped at 16. In the CROWNS study, 82% of practitioners enrolled 20 or more patients (capped at 22); in the PREDICT study, 58% enrolled less than 10 patients, and some (the endodontists) enrolled as many as 50 patients.

**Table 4.** Characteristics of practitioners and patients participating in a prospective clinical study conducted by the National Dental Practice-Based Research Network 2012–2019

	Practitioners			Patients	
	(*N* = 591)			(*N* = 12,159)	
	*N*	Col %		*N*	Col %
Gender			Gender		
Male	413	70%	Male	4,249	35%
Female	174	30%	Female	7,897	65%
Age in 2014			Age at enrollment		
<45 years	211	36%	<35 years	2,140	18%
45 to 54 years	120	21%	35 to 44 years	2,032	17%
55 to 64 years	210	36%	45 to 54 years	2,649	22%
65 or more years	44	8%	55 to 64 years	2,856	24%
			65 or more years	2,200	19%
	mean = 50 (sd = 11); median = 52 (IQR:40-59); range: 24–77			mean = 50 (sd = 15); median = 52 (IQR: 39–62) range: (18–100)	
Race^1^-ethnicity			Race^1^-ethnicity		
White	456	78%	White	9,516	79%
African-American/Black	27	5%	African-American/Black	818	7%
Asian	69	12%	Asian	341	3%
Hispanic	16	3%	Multi/other	863	7%
Other	16	3%	Hispanic	437	4%
Practice type			Education level attained		
Solo private practice	278	48%	High school graduate/GED	1,882	16%
Owner, private practice	146	25%	Some college/AD	4,155	35%
Associate, private practice	63	11%	Bachelor degree	3,512	29%
HP/PDA/Other PPO^2^	51	9%	Graduate degree	2,457	20%
Public/Federal	11	2%			
Academic	31	5%			
			Any dental insurance		
General/Specialist			No	2,521	21%
General	499	85%	Yes	9,638	79%
Specialist	91	15%			
Total number of patients enrolled					
1 – 9	141	25%			
10 – 19	141	25%			
20 – 29	160	28%			
30+	131	23%			
	mean = 21 (sd = 15); median = 20 (IQR:10-27); range: 1–76				

1 All races listed are non-Hispanic.

2 PPO: Preferred provider organization, HP:Health Partners, PDA: Permanente Dental Associates.

### Patient characteristics

A total of 12,159 patients participated in one of the five studies (no patient participated in more than one study). In contrast to practitioners, the majority (65%) of patients were female (Table 4). Patients’ age and race distributions were nearly the same as those of practitioners, viz., patients’ mean age was 50 (*sd* = 15), and 79% were non-Hispanic White. Around half of the patients had a bachelor’s degree or higher, and 79% had some dental insurance. These characteristics were similar across studies. All studies had patients from each of the Network’s six geographic/administrative regions.

### Composite participation/follow-up measures

**Figure 2.**
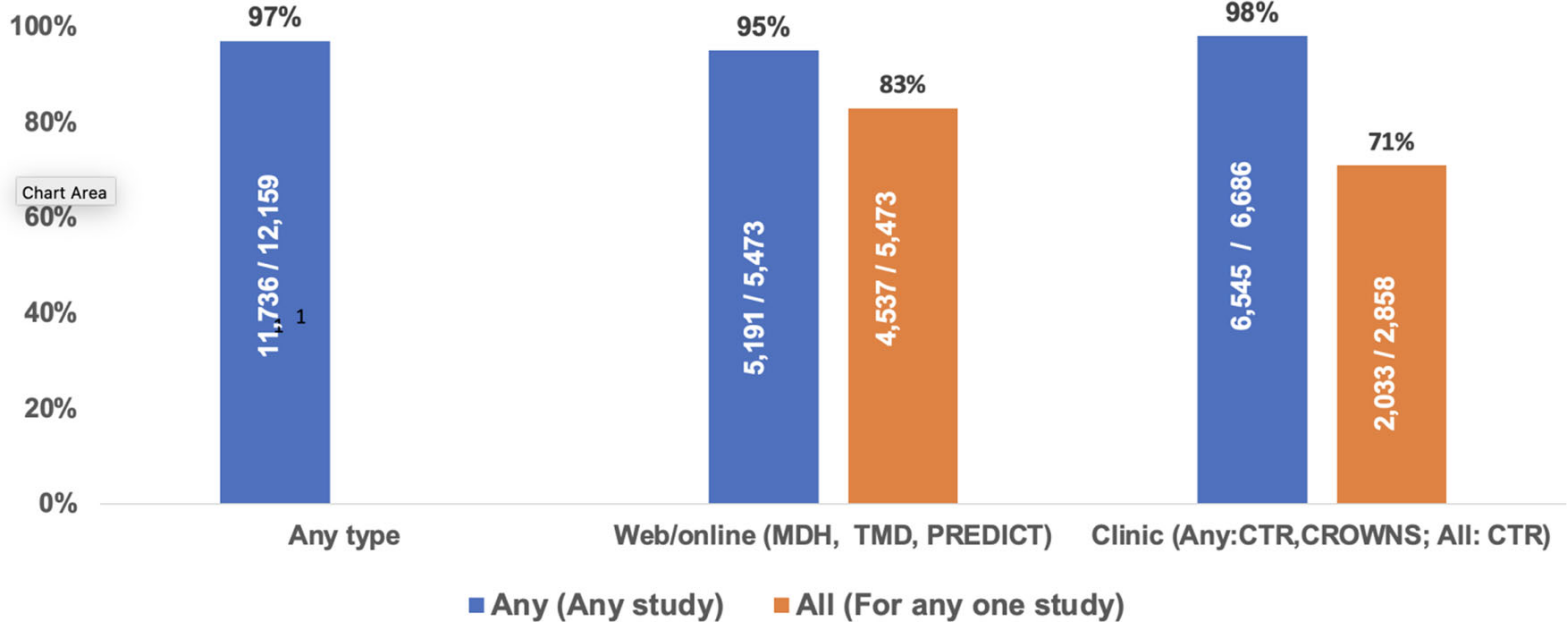
Composite participation measures. CROWNS: factors for successful crowns, CTR: Cracked Tooth Registry, MDH: management of dentin hypersensitivity, PREDICT: predicting outcomes of root canal treatment, TMD: temporomandibular disorders.

Overall, 97% (11,736/12,159) of the patients participated in at least one follow-up activity, either online or clinic visits (Fig. 2). Of the three studies (MDH, TMD, PREDICT) for which online follow-up completion was requested, 95% (5,191/5,473) of patients completed at least one and 83% (4,537/5,473) completed all for at least one study. Of the two studies (CTR, CROWNS) requiring a clinic visit, 98% (6,545/6,686) completed at least one; 71% (2,033/2,858) of CTR patients completed all three recall visits.

### Associations with patient retention (return for recall visits, completing post-visit online assessments) were also analyzed

The numbers are so large for any participation that minor differences (not clinically meaningful) are statistically significant, e.g., patients of general practitioners compared to specialists (97% vs. 95%, *P* = .003) and female compared to male patients (97% vs. 96%, *P* < .001). Because of this, we only describe the associations with attending all clinic visits (CTR only) or completing all online (MDH, TMD, or PREDICT) assessments (Table 5). For clinic visits, at the practitioner level, patients of public, federal, or academic practices were more likely to return for all recall visits than patients of preferred provider organizations, and of practitioners who had enrolled more patients compared to fewer patients (Table 6), At the patient level, older patients were more likely to return for all visits than were younger patients. Associations with completing online forms differed from those for clinic visits. The only difference at the practitioner level was race-ethnicity, viz., patients of non-Hispanic white or Asian or Hispanic practitioners were more likely to complete online forms than were patients of African-American, multi-racial, or other unspecified races. At the patient level, females, older, more educated, and (as with practitioners) non-Hispanic white or Asian and Hispanic completed online follow-up more than African-American, multi-racial, and other unspecified races completed post-visit online assessments more frequently.

**Table 5.** Characteristics of practitioners and patients participating in specified studies^1^ conducted by the National Dental Practice-Based Research Network 2012–2019

	Clinic follow-up, all visits, CTR			ALL web follow-up for any study: MDH, TMD, PREDICT		
	(*N* = 2,858)			(*N* = 5,473)		
	All follow-up visits			All web follow-up		
	All	*N*	Row %	All	*N*	Row %
**Practitioner characteristics**						
**Dentist Gender**						
Male	2,070	1,462	71%	3,282	2,728	83%
Female	785	571	73%	1,901	1,564	82%
		P^4^ = 0.3			*P* = 0.4	
Dentist Age in 2014						
<45 years	635	409	64%	1,971	1,604	81%
45 to 54 years	612	440	72%	978	826	84%
55 to 64 years	1,401	1,051	75%	1,850	1,557	84%
65 or more years	193	118	61%	316	247	78%
		*P* < 0.001			*P* = 0.008	
Dentist race^2^-ethnicity						
White	2,420	1,747	72%	3,918	3,300	84%
African-American/Black	96	62	65%	256	166	65%
Asian	219	148	68%	635	532	84%
Hispanic	54	40	74%	197	161	82%
Other	69	36	52%	111	76	68%
		*P* = 0.002			*P* < 0.001	
Practice type						
Solo private practice	1,706	1,250	73%	1,365	1,080	79%
Owner, private practice	635	476	75%	2,327	1,967	84%
Associate, private practice	216	108	50%	569	493	84%
HP/PDA/Other PPO^3^	175	100	57%	371	325	88%
Public/Federal	26	21	81%	113	79	70%
Academic	67	60	90%	347	300	86%
		*P* < 0.001			P = <0.001	
General/Specialist						
General	2,828	2,017	71%	3,858	3,173	82%
Specialist	30	16	53%	1,322	1,121	85%
		*P* = 0.03			*P* = 0.03	
Total number of patients enrolled						
1 – 9	327	191	58%	1,171	946	81%
10 – 19	1,731	1,184	68%	2,892	2,398	83%
20 - 29	800	658	82%	890	745	84%
30+	0	0		423	363	86%
		*P* < 0.001			*P* = 0.09	
**Patient characteristics**						
**Gender**						
Male	1,044	757	72%	1,598	1,171	78%
Female	1,813	1,275	70%	3,953	3,355	85%
		*P* = 0.2			*P* < 0.001	
Age at study enrollment						
< 35 years	166	116	70%	1,567	1,279	82%
35 to 44 years	389	255	66%	1,118	903	81%
45 to 54 years	750	549	73%	1,129	938	83%
55 to 64 years	841	661	79%	968	826	85%
65 or more years	470	344	73%	651	563	86%
		*P* < 0.001			*P* = 0.004	
Race^2^-ethnicity						
White	2,394	1,724	72%	4,069	3,461	85%
African-American/Black	134	86	64%	424	311	73%
Asian	48	27	56%	183	152	83%
Multi/other	57	45	79%	550	422	77%
Hispanic	186	123	66%	164	132	80%
		*P* = 0.01			*P* < 0.001	
Education level attained						
High school graduate/GED	407	299	73%	836	631	75%
Some college/AD	950	657	69%	1,993	1,634	82%
Bachelor degree	879	630	72%	1,521	1,307	86%
Graduate degree	603	438	73%	1,054	919	87%
		*P* = 0.3			*P* < 0.001	
Any dental insurance						
No	645	460	71%	1,061	841	79%
Yes	2,213	1,573	71%	4,412	3,696	84%
		*P* = 0.9			*P* < 0.001	

1 CTR = Cracked Tooth Registry; CROWNs = Factors for successful crowns; MDH = Management of dentin hypersensitivity; TMD = Management of painful temporomandibular disorders; PREDICT = Predicting outcomes of root canal treatment.

2 All races listed are non-Hispanic.

3 PPO = Preferred provider organization; HP = Health Partners; PDA = Permanente Dental Associates.

4 P: From chi-squared statistic.

**Table 6.** Associations of practitioner and patient characteristics with attending or completing all requested follow-up visits/assessments for specified studies^1^

	Individual^2^		Full model^3^		
	Odds Ratio	p	Odds Ratio	95% Confidence Interval	p
**All follow-up clinic visits: CTR**					
**Practitioner characteristics**					
Female vs. male	1.18	0.4	1.14	0.71–1.82	0.6
Age (per 10 years)	1.14	0.2	1.03	0.80–1.31	0.8
Race-ethnicity	cat^4^	0.5	cat		0.9
Practice type	cat	0.009	cat		0.014
Specialist vs. general practitioner	0.34	0.4	0.32	0.04–2.49	0.3
Number of patients enrolled (per 10)	1.92	< 0.001	1.55	1.15–2.08	0.005
**Patient characteristics**					
Female vs. male	0.88	0.08	0.87	0.73–1.04	0.12
Age (per 10 years)	1.10	0.005	1.10	1.02–1.19	0.014
Race-ethnicity	cat	0.09	cat		0.10
Bachelor degree or higher	1.04	0.6	1.01	0.86–1.20	0.9
Any vs. no dental insurance	1.01	0.9	1.07	0.87–1.31	0.5
**All follow-up web visits: MDH, TMD, PREDICT**					
**Practitioner characteristics**					
Female vs. male	0.92	0.5	0.95	0.76–1.18	0.6
Age (per 10 years)	1.02	0.7	1.00	0.89–1.13	0.9
Race-ethnicity	cat	0.01	cat		0.03
Practice type	cat	0.008	cat		0.3
Specialist vs. general practitioner	1.26	0.04	1.11	0.87–1.41	0.4
Number of patients enrolled (per 10)	1.10	0.2	1.05	0.92–1.19	0.5
**Patient characteristics**					
Female vs. male	1.50	< 0.001	1.56	1.30–1.87	< 0.001
Age (per 10 years)	1.09	0.001	1.06	1.01–1.12	0.03
Race-ethnicity	cat	0.003	cat		0.02
Bachelor degree or higher	1.49	< 0.001	1.36	1.17–1.60	< 0.001
Any vs. no dental insurance	1.26	0.02	1.18	0.97–1.44	0.1

1 CTR = Cracked Tooth Registry; CROWNs = Factors for successful crowns; MDH: Management of dentin hypersensitivity; TMD = Management of painful temporomandibular disorders; PREDICT = Predicting outcomes of root canal treatment.

2 Individual: Adjusted only for patients clustered within practice using generalized estimating equations.

3 Full model: Includes all characteristics listed.

4 Cat: categorical.

## Discussion

The Network used various methods to recruit and retain dental practitioners and patients in clinical studies. Practitioner demographics were similar across studies, e.g., the majority were non-Hispanic white males, with mean ages of 50–53 years. The mean number of patients each practitioner recruited varied from 10–19 across the studies. Patient demographics were also similar across studies, e.g., the majority were non-Hispanic white females, had some dental insurance, mean ages 43–55 years, and 46% to 52% had a bachelor’s degree or higher. These practitioner demographics are consistent with the characteristics of dentists nationally and of patients who enter the dental care system for treatment [27].

Our five studies achieved excellent retention rates at both the practitioner (mainly in the CTR study) and patient levels. “Any FU” rates varied from 91% to 96.5%, and rates for participating in all assessments ranged from 68% to 87%. A 3-year study to examine the outpatient management of acute low back pain showed that by the end of the second year of FU, 13 of these 41 clinicians (32%) moved on to other practices, resulting in their study retention rate of 68% [28]. In contrast, our CTR study showed 209 practitioners who enrolled patients; 195 (93%) continued participating for 3 years. The Network has achieved these high retention rates for clinical studies by drawing from its experiences and strategies cited in the literature [29–31]. The Network retains practitioners and patients by developing informal and professional relationships through continuous and positive communication and offers financial incentives to encourage their continued participation. As with any research network, it was vital for the Network to establish a continuously updated database that helps with effective communication [28].

For all the studies, the recruitment of patients was limited to 6-12 months, so the practitioner would not be involved in study activities for extended durations and possibly tire of it [14]. The visit recall periods were carefully strategized, largely so that the visits were more likely to fit on a schedule consistent with patient recall patterns and insurance compensation. Successful clinical studies rely on recruiting and retaining an adequate number of participants [2]. For the CTR study, the association with the retention of patients was practitioners with a greater number of enrolled patients. This was true regardless of the number or type of FU assessments (1–3). These practitioners could be considered more invested in the study and more effectively communicate the value of the study to their patients.

When joining a study, a patient’s literacy level, sociocultural background, and knowledge of clinical research play a vital role in their decision-making process [2]. Similarly, our results reflected greater participation in post-visit online assessments by patients who were female, older, or more educated. Digital tools, such as remote meetings and online assessments, were more strongly associated with higher practitioner/patient retention rates. This can be attributed to relieving the patients of the burden of attending in-person visits and travel time that could lead to withdrawal from the study [2]. Findings in a similar study about retention and recruitment strategies of young women reported that email announcements and reminders were associated with greater numbers of participants and FU completions [32].

This report has limitations. First, the studies were conducted within an existing PBRN, and many practitioner recruitment methods can only be used in such networks. Other PBRNs could incorporate methods described in this report for future clinical studies. All the methods described are from observational studies, none of which had a randomized component to evaluate the effectiveness of any particular recruitment or retention strategy. We cannot state whether any specific method is effective except by our impression and interactions with practitioners. Finally, like most studies, those described in this report were funded by a federal agency to conduct the described study but not to conduct any associated methodological study. This report also has strengths. Several studies were described, each involving over 100 practitioners and over 1,000 patients, and were conducted nationwide (US). No substantial regional differences were observed. In addition, participation methods included electronic and in-person.

### Implications for research

The study analysis showcases that the Network’s recruitment and retention strategies yielded favorable practitioner and patient involvement outcomes. These findings inform the broader research community about optimizing recruitment and retention strategies for clinical studies in community-based practices. The study underscores the significance of considering practitioner and patient characteristics when designing such strategies, ultimately contributing to more effective and efficient research initiatives in community-based settings.

## Conclusion

These five studies demonstrate overall efficiency in enrolling practitioners and patients by adopting the Network’s recruitment and retention strategies. The phased approach for practitioner recruitment included live events and direct communication, whereas retention involved strong relationships, periodic contact, and a continuously updated practitioner database. Similarly, patient recruitment utilized diverse strategies like strategically placed flyers, targeted chart reviews, and personal discussions during appointments. Patient incentives and active engagement of dental office staff enhanced patient participation, with clear communication and tracking procedures ensuring successful follow-up visits and the impact of National Dental PBRN studies. This report offers how practitioner and patient characteristics might enable effective recruitment and retention strategies; this may be useful when planning studies that seek to maximize recruitment and retention.

## Supporting information

Mungia et al. supplementary material Mungia et al. supplementary material

## Supplementary material

The supplementary material for this article can be found at https://doi.org/10.1017/cts.2024.499.
