# Supplementary material for: Recruitment strategies and retention rates for five National Dental PBRN studies
Source: J Clin Transl Sci. 2024 Mar 22;8(1):e56. doi: 10.1017/cts.2024.499 (PMC11010183; doi:10.1017/cts.2024.499)
Supplement: Mungia et al. supplementary material [file S2059866124004990sup001.docx]

**SUPPLEMENTAL TABLES**

Supp Table 1. Characteristics of practitioners and patients participating in specified studies^1^ conducted by the National Dental Practice-Based Research Network 2012-2019

Supp Table 2. Associations of practitioner and patient characteristics with attending or completing any requested follow-up visits/assessments for specified studies^1^.

Supp Table 3. Characteristics of practitioners participating in a prospective clinical study^1^ conducted by the National Dental Practice-Based Research Network 2012-2019

Supp Table 4. Characteristics of patients participating in a prospective clinical study^1^ conducted by the National Dental Practice-Based Research Network 2012-2019

Supp Table 5. Associations of practitioner and patient characteristics with attending or completing any follow-up (FU) visits/assessments and with attending/completing all follow-up assessments for each study.

**Supp Table 1. Characteristics of practitioners and patients participating in specified studies^1^ conducted by the National Dental Practice-Based Research Network 2012-2019**

|  | **Any clinic follow-up (CTR, CROWNS)** | | |  | **Any web follow-up (MDH, TMD, PREDICT)** | | |
| --- | --- | --- | --- | --- | --- | --- | --- |
|  | (N = 6,686) | | |  | (N = 5,473) | | |
|  |  | Any clinic follow-up | |  |  | Any web follow-up | |
|  | All | N | Row % |  | All | N | Row % |
| **Practitioner characteristics** |  |  |  |  |  |  |  |
| Practitioner gender |  |  |  |  |  |  |  |
| Male | 4,863 | 4,754 | 98% |  | 3,282 | 3,117 | 95% |
| Female | 1,761 | 1,732 | 98% |  | 1,901 | 1,801 | 95% |
|  |  | P^4^ = .1 | |  |  | P = .7 | |
| Practitioner age in 2014 |  |  |  |  |  |  |  |
| <45 years | 2,021 | 1,979 | 98% |  | 1,971 | 1,861 | 94% |
| 45 to 54 years | 1,338 | 1,307 | 98% |  | 978 | 928 | 95% |
| 55 to 64 years | 2,837 | 2,773 | 98% |  | 1,850 | 1,768 | 96% |
| 65 or more years | 453 | 450 | 99% |  | 316 | 297 | 94% |
|  |  | P = .15 | |  |  | P = .4 | |
| Practitioner race^2^-ethnicity |  |  |  |  |  |  |  |
| White | 5,443 | 5,333 | 98% |  | 3,918 | 3,749 | 96% |
| African-American/Black | 294 | 285 | 97% |  | 256 | 214 | 84% |
| Asian | 581 | 563 | 97% |  | 635 | 611 | 96% |
| Hispanic | 180 | 176 | 98% |  | 197 | 188 | 95% |
| Other | 169 | 169 | 100% |  | 111 | 94 | 85% |
|  |  | P = .1 | |  |  | P < .001 | |
| Practice type |  |  |  |  |  |  |  |
| Solo private practice | 3,669 | 3,606 | 98% |  | 1,365 | 1,276 | 93% |
| Owner, private practice | 1,627 | 1,581 | 97% |  | 2,327 | 2,234 | 96% |
| Associate, private practice | 529 | 516 | 98% |  | 589 | 560 | 95% |
| HP/PDA/Other PPO^3^ | 573 | 559 | 98% |  | 371 | 361 | 97% |
| Public/Federal | 74 | 73 | 99% |  | 113 | 99 | 88% |
| Academic | 90 | 90 | 100% |  | 347 | 329 | 95% |
|  |  | P = .09 | |  |  | P <.001 | |
| General/Specialist |  |  |  |  |  |  |  |
| General | 6,596 | 6,459 | 98% |  | 3,858 | 3,659 | 95% |
| Specialist | 71 | 67 | 94% |  | 1,322 | 1,260 | 95% |
|  |  | P = .06 | |  |  | P = .5 | |
|  |  |  |  |  |  |  |  |
| Total number of patients enrolled |  |  |  |  |  |  |  |
| 1 – 9 | 428 | 394 | 92% |  | 1,171 | 1,097 | 94% |
| 10 – 19 | 2,281 | 2,220 | 97% |  | 2,892 | 2,759 | 95% |
| 20 - 29 | 3,977 | 3,931 | 99% |  | 890 | 834 | 94% |
| 30+ | 0 | 0 |  |  | 423 | 407 | 96% |
|  |  | P < .001 | |  |  | P = .03 | |
|  |  |  |  |  |  |  |  |
| **Patient characteristics** |  |  |  |  |  |  |  |
| Gender |  |  |  |  |  |  |  |
| Male | 2,741 | 2,675 | 98% |  | 1,508 | 1,389 | 92% |
| Female | 3,944 | 3,869 | 98% |  | 3,953 | 3,791 | 96% |
|  |  | P = .16 | |  |  | P < .001 | |

| Supp-Table 1 - continued |  |  |  |  |  |  |  |
| --- | --- | --- | --- | --- | --- | --- | --- |
|  |  |  |  |  |  |  |  |
| Age at study enrollment |  |  |  |  |  |  |  |
| < 35 years | 573 | 560 | 98% |  | 1,567 | 1,475 | 94% |
| 35 to 44 years | 914 | 901 | 98% |  | 1,118 | 1,044 | 93% |
| 45 to 54 years | 1,520 | 1,503 | 99% |  | 1,129 | 1,080 | 96% |
| 55 to 64 years | 1,888 | 1,866 | 99% |  | 968 | 929 | 96% |
| 65 or more years | 1,549 | 1,535 | 99% |  | 651 | 629 | 97% |
|  |  | P = .14 | |  |  | P = .005 | |
|  |  |  |  |  |  |  |  |
| Race^2^-ethnicity |  |  |  |  |  |  |  |
| White | 5,447 | 5,336 | 98% |  | 4,069 | 3,913 | 96% |
| African-American/Black | 394 | 383 | 97% |  | 424 | 382 | 91% |
| Asian | 158 | 157 | 99% |  | 183 | 175 | 96% |
| Multi/other | 313 | 300 | 96% |  | 550 | 497 | 90% |
| Hispanic | 273 | 272 | 99% |  | 164 | 152 | 93% |
|  |  | P = .01 | |  |  | P < .001 | |
| Education level attained |  |  |  |  |  |  |  |
| High school graduate/GED | 1,046 | 1,016 | 97% |  | 836 | 755 | 90% |
| Some college/AD | 2,162 | 2,113 | 98% |  | 1,993 | 1,893 | 95% |
| Bachelor degree | 1,991 | 1,959 | 98% |  | 1,521 | 1,472 | 97% |
| Graduate degree | 1,403 | 1,376 | 98% |  | 1,054 | 1,013 | 96% |
|  |  | P = .12 | |  |  | P < .001 | |
| Any dental insurance |  |  |  |  |  |  |  |
| No | 1,460 | 1,430 | 98% |  | 1,061 | 986 | 93% |
| Yes | 5,226 | 5,115 | 98% |  | 4,412 | 4,205 | 95% |
|  |  | P = .9 | |  |  | P = .002 | |

^1^CTR: Cracked Tooth Registry; CROWNs: Factors for successful crowns; MDH: Management of dentin hypersensitivity; TMD: Management of painful temporomandibular disorders; PREDICT: Predicting outcomes of root canal treatment

^2^All races listed are non-Hispanic

^3^PPO: Preferred provider organization, HP:Health Partners, PDA: Permanente Dental Associates

^4^P: From chi-squared statistic

**Supp Table 2. Associations of practitioner and patient characteristics with attending or completing any requested follow-up visits/assessments for specified studies^1^.**

|  | **Individual^2^** | |  | **Full^3^ model** | | |
| --- | --- | --- | --- | --- | --- | --- |
|  | **Odds Ratio** | **p** |  | **Odds Ratio** | **95% Confidence Interval** | **p** |
| **Any clinic follow-up visits: CTR, CROWN** |  |  |  |  |  |  |
| **Practitioner characteristics** |  |  |  |  |  |  |
| Female vs. male | 1.78 | .2 |  | 0.66 | 0.28 - 1.57 | .4 |
| Age (per 10 years) | 1.10 | .6 |  | 1.40 | 1.02 - 1.90 | .04 |
| Non-Hispanic white | 1.03 | .9 |  | 1.27 | 0.46 - 3.51 | .8 |
| Private practice | 1.38 | .6 |  | 0.65 | 0.23 - 1.83 | .4 |
| Specialist vs. general practitioner | 0.61 | .5 |  | 0.24 | 0.07 - 0.85 | .03 |
| Number of patients enrolled (per 10) | 1.83 | .07 |  | 1.01 | 0.53 - 1.90 | .9 |
| **Patient characteristics** |  |  |  |  |  |  |
| Female vs. male | 1.31 | .014 |  | 2.11 | 1.42 - 3.14 | .003 |
| Age (per 10 years) | 1.14 | .2 |  | 1.06 | 0.88 - 1.28 | .5 |
| Non-Hispanic white | 0.88 | .4 |  | 1.27 | 0.71 - 2.29 | .4 |
| Bachelor degree or higher | 1.19 | .2 |  | 1.42 | 0.82 - 2.46 | .2 |
| Any vs. no dental insurance | 0.99 | .9 |  | 0.72 | 0.39 - 1.32 | .3 |
|  |  |  |  |  |  |  |
|  | |  |  |  |  |  |
| **ANY follow-up web visits: MDH, TMD, PREDICT** | |  |  |  |  |  |
| **Practitioner characteristics** |  |  |  |  |  |  |
| Female vs. male | 0.94 | 0.7 |  | 1.01 | 0.72 - 1.41 | .9 |
| Age (per 10 years) | 1.06 | .5 |  | 1.05 | 0.88 - 1.24 | .6 |
| Race-ethnicity | Cat^4^ | .02 |  | cat |  | .001 |
| Practice type | cat | .06 |  | cat |  | .2 |
| Specialist vs. general practitioner | 1.15 | .4 |  | 1.01 | 0.72 - 1.41 | .9 |
| Number of patients enrolled (per 10) | 1.04 | .7 |  | 1.01 | 0.82 - 1.25 | .9 |
| **Patient characteristics** |  |  |  |  |  |  |
| Female vs. male | 1.81 | <.001 |  | 1.96 | 1.46 - 2.62 | <.001 |
| Age (per 10 years) | 1.16 | .003 |  | 1.12 | 1.01 - 1.24 | .04 |
| Race-ethnicity | cat | .003 |  | cat |  | .02 |
| Bachelor degree or higher | 1.75 | <.001 |  | 1.43 | 1.09 - 1.87 | .01 |
| Any vs. no dental insurance | 1.40 | .04 |  | 1.18 | 0.87 - 1.60 | .3 |

^1^CTR: Cracked Tooth Registry; CROWNs: Factors for successful crowns; MDH: Management of dentin hypersensitivity; TMD: Management of painful temporomandibular disorders; PREDICT: Predicting outcomes of root canal treatment

^2^Individual: Adjusted only for patients clustered within practice using generalized estimating equations (GEE)

^3^Full model: Includes all characteristics listed

^4^Cat: categorical

**Supp Table 3. Characteristics of practitioners participating in a prospective clinical study^1^ conducted by the National Dental Practice-Based Research Network 2012-2019**

|  | **CTR** | | **CROWNS** | | **MDH** | | **TMD** | | **PREDICT** | |
| --- | --- | --- | --- | --- | --- | --- | --- | --- | --- | --- |
|  | (N =209) | | (N =205) | | (N =162^2^) | | (N =182^2^) | | (N =140^2^) | |
|  | N | Column % | N | Column % | N | Column % | N | Column % | N | Column % |
| Gender |  |  |  |  |  |  |  |  |  |  |
| Male | 153 | 74% | 148 | 73% | 100 | 62% | 113 | 62% | 102 | 73% |
| Female | 55 | 26% | 54 | 27% | 62 | 38% | 68 | 38% | 38 | 27% |
|  |  |  |  |  |  |  |  |  |  |  |
| Age at study enrollment |  |  |  |  |  |  |  |  |  |  |
| <45 years | 50 | 24% | 68 | 33% | 51 | 31% | 71 | 39% | 48 | 34% |
| 45 to 54 years | 43 | 21% | 41 | 20% | 32 | 20% | 32 | 18% | 26 | 19% |
| 55 to 64 years | 98 | 47% | 73 | 36% | 65 | 40% | 56 | 31% | 52 | 37% |
| 65 or more years | 17 | 8% | 22 | 11% | 14 | 9% | 21 | 12% | 14 | 10% |
| Descriptive age statistics^3^ | mean =53  (sd=10) median=56  (IQR:45-60)  range:27-73 | | mean =52  (sd=11) median=54  (IQR:41-61)  range:30-79 | | mean =51  (sd=11) median=54  (IQR:41-60)  range:27-73 | | mean =50  (sd=12) median=50  (IQR:40-60)  range:27-73 | | mean =52  (sd=12) median=53  (IQR:42-62)  range:27-73 | |
| Race^4^-ethnicity |  |  |  |  |  |  |  |  |  |  |
| White | 173 | 84% | 161 | 77% | 128 | 79% | 135 | 74% | 100 | 73% |
| African-American/Black | 10 | 5% | 11 | 5% | 7 | 4% | 4 | 2% | 11 | 8% |
| Asian | 16 | 8% | 20 | 10% | 16 | 10% | 29 | 16% | 15 | 11% |
| Hispanic | 7 | 3% | 7 | 3% | 6 | 4% | 7 | 4% | 6 | 4% |
| Other | 0 | 0% | 5 | 2% | 5 | 3% | 4 | 2% | 5 | 4% |
|  |  |  |  |  |  |  |  |  |  |  |
| Practice type |  |  |  |  |  |  |  |  |  |  |
| Solo private practice | 118 | 58% | 104 | 52% | 73 | 46% | 74 | 42% | 59 | 43% |
| Owner, private practice | 46 | 22% | 51 | 26% | 43 | 27% | 48 | 27% | 41 | 30% |
| Associate, private practice | 17 | 8% | 19 | 10% | 19 | 12% | 20 | 11% | 14 | 10% |
| Preferred provider organization | 16 | 8% | 20 | 10% | 17 | 11% | 11 | 6% | 12 | 9% |
| Other | 8 | 4% | 6 | 3% | 8 | 5% | 25 | 14% | 11 | 8% |
|  |  |  |  |  |  |  |  |  |  |  |
| General/Specialist |  |  |  |  |  |  |  |  |  |  |
| General | 207 | 99% | 202 | 99% | 152 | 94% | 147 | 81% | 100 | 71% |
| Specialist | 2 | 1% | 2 | 1% | 10 | 6% | 34 | 19% | 40 | 29% |
|  |  |  |  |  |  |  |  |  |  |  |
| Number of patients enrolled |  |  |  |  |  |  |  |  |  |  |
| 1 – 9 | 54 | 26% | 15 | 7% | 64 | 40% | 93 | 51% | 81 | 58% |
| 10 – 14 | 52 | 25% | 10 | 5% | 26 | 16% | 35 | 19% | 23 | 16% |
| 15 – 19 | 63 | 30% | 11 | 5% | 72 | 44% | 17 | 9% | 16 | 11% |
| 20+ | 40 | 19% | 169 | 82% | 0 | 0% | 37 | 20% | 20 | 14% |
| Descriptive statistics for number  of patients enrolled | mean =14  (sd=5) median=14  (IQR:9-19)  range: 1-20 | | mean =19  (sd=4) median=20  (IQR:20-20)  range: 1-22 | | mean =11  (sd=5) median=12  (IQR:6-16)  range: 1-16 | | mean =10  (sd=6) median=9  (IQR:5-17)  range: 1-22 | | mean =11  (sd=10) median=7  (IQR:4-15)  range: 1-50 | |

^1^CTR: Cracked Tooth Registry; CROWNs: Factors for successful crowns; MDH: Management of dentin hypersensitivity; TMD: Management of painful temporomandibular disorders; PREDICT: Predicting outcomes of root canal treatment

^2^Missing practitioner enrollment questionnaire data (primarily demographics) for 9 MDH practitioners, 3 TMD, and 13 PREDICT practitioners.

^3^sd: standard deviation; IQR: Inter-quartile range

^4^Racial groups are non-Hispanic

**Supp Table 4. Characteristics of patients participating in a prospective clinical study^1^ conducted by the National Dental Practice-Based Research Network 2012-2019**

|  | **CTR** | | **CROWNS** | | **MDH** | | **TMD** | | **PREDICT** | |
| --- | --- | --- | --- | --- | --- | --- | --- | --- | --- | --- |
|  | (N =2,858) | | (N =3,806) | | (N =1,868) | | (N =1,886) | | (N =1,719) | |
|  | N | Column % | N | Column % | N | Column % | N | Column % | N | Column % |
| Gender |  |  |  |  |  |  |  |  |  |  |
| Male | 1,044 | 37% | 1,697 | 44% | 488 | 26% | 320 | 17% | 700 | 41% |
| Female | 1,813 | 63% | 2,131 | 56% | 1,380 | 74% | 1,562 | 83% | 1,011 | 59% |
|  |  |  |  |  |  |  |  |  |  |  |
| Age at study enrollment |  |  |  |  |  |  |  |  |  |  |
| < 35 years | 197 | 6% | 407 | 11% | 522 | 28% | 647 | 34% | 398 | 23% |
| 35 to 44 years | 439 | 15% | 525 | 14% | 424 | 23% | 401 | 21% | 293 | 17% |
| 45 to 54 years | 812 | 28% | 770 | 20% | 450 | 24% | 333 | 18% | 346 | 20% |
| 55 to 64 years | 912 | 32% | 1047 | 27% | 326 | 18% | 275 | 15% | 367 | 22% |
| 65 or more years | 497 | 17% | 1,079 | 28% | 139 | 8% | 221 | 12% | 291 | 17% |
| Descriptive age statistics^2^ | mean =54  (sd=12)  median=55  (IQR:46-62)  range:19-85 | | mean =55  (sd=15)  median=56  (IQR:45-66)  range:18-100 | | mean =44  (sd=14)  median=44  (IQR:33-54)  range:18-87) | | mean =43  (sd=16)  median=42  (IQR:30-55)  range:18-86 | | mean =48  (sd=16)  median=49  (IQR:36-61)  range:18-94 | |
|  |  |  |  |  |  |  |  |  |  |  |
| Race^3^-ethnicity |  |  |  |  |  |  |  |  |  |  |
| White | 2,394 | 85% | 3,053 | 81% | 1,445 | 79% | 1,451 | 78% | 1,173 | 70% |
| African-American/Black | 134 | 5% | 260 | 7% | 136 | 7% | 82 | 4% | 206 | 12% |
| Asian | 48 | 2% | 110 | 3% | 60 | 3% | 64 | 3% | 59 | 4% |
| Hispanic | 186 | 7% | 256 | 7% | 151 | 8% | 212 | 11% | 187 | 11% |
| Other | 57 | 2% | 87 | 2% | 48 | 3% | 63 | 3% | 53 | 3% |
|  |  |  |  |  |  |  |  |  |  |  |
| Education level attained |  |  |  |  |  |  |  |  |  |  |
| High school graduate/GED | 407 | 14% | 639 | 17% | 273 | 15% | 253 | 13% | 310 | 19% |
| Some college/AD | 950 | 34% | 1,212 | 32% | 720 | 39% | 677 | 36% | 596 | 36% |
| Bachelor’s degree | 879 | 31% | 1,112 | 30% | 510 | 27% | 567 | 30% | 444 | 26% |
| Graduate degree | 603 | 21% | 800 | 21% | 353 | 19% | 373 | 20% | 528 | 20% |
|  |  |  |  |  |  |  |  |  |  |  |
| Any dental insurance |  |  |  |  |  |  |  |  |  |  |
| No | 645 | 23% | 815 | 21% | 384 | 21% | 257 | 14% | 420 | 24% |
| Yes | 2,213 | 77% | 3,013 | 79% | 1,484 | 79% | 1,629 | 86% | 1,299 | 76% |
|  |  |  |  |  |  |  |  |  |  |  |
| Network Region |  |  |  |  |  |  |  |  |  |  |
| Western | 420 | 15% | 560 | 15% | 217 | 12% | 212 | 11% | 167 | 10% |
| Midwest | 386 | 13% | 638 | 17% | 331 | 19% | 456 | 24% | 396 | 23% |
| Southwest | 535 | 19% | 686 | 18% | 271 | 15% | 375 | 20% | 423 | 25% |
| South Central | 553 | 19% | 884 | 23% | 328 | 18% | 290 | 15% | 303 | 18% |
| South Atlantic | 487 | 17% | 602 | 16% | 289 | 16% | 241 | 13% | 248 | 14% |
| Northeast | 477 | 17% | 437 | 11% | 335 | 19% | 312 | 16% | 182 | 11% |

^1^CTR: Cracked Tooth Registry; CROWNs: Factors for successful crowns; MDH: Management of dentin hypersensitivity; TMD: Management of painful temporomandibular disorders; PREDICT: Predicting outcomes of root canal treatment

^2^sd: standard deviation; IQR: Inter-quartile range

^3^Racial groups are non-Hispanic

**Supp Table 5. Associations of practitioner and patient characteristics with attending or completing any follow-up (FU) visits/assessments and with attending/completing all follow-up assessments for each study.**

|  | **Individual^1^** | | **Full^2^ model** | | **Final Reduced^3^ model** | | |
| --- | --- | --- | --- | --- | --- | --- | --- |
| **Practitioner and patient characteristics** | **Odds Ratio** | **p** | **Odds Ratio** | **P** | **Odds Ratio** | **95% Confidence interval** | **p** |
| **Cracked tooth registry (CTR)** |  |  |  |  |  |  |  |
| Any FU |  |  |  |  |  |  |  |
| Dentist age (per 10 yrs^4^) | 1.29 | 0.06 | 1.17 | 0.2 | w^9^ |  |  |
| # patients (per 5) | 1.81 | <0.001 | 1.78 | <0.001 | 1.84 | 1.41 - 2.41 | <0.001 |
| Pt^5^ age (per 10 yrs) | 1.22 | 0.002 | 1.24 | 0.003 | 1.24 | 1.09 - 1.42 | 0.002 |
| Pt race-ethnicity | cat^8^ | 0.09 | cat | 0.086 | w |  |  |
|  |  |  |  |  |  |  |  |
| All FU |  |  |  |  |  |  |  |
| Dentist age | cat | 0.013 | cat | 0.5 | w |  |  |
| Practice type | cat | 0.004 | cat | 0.007 | cat |  | 0.002 |
| # patients (per 5) | 1.51 | <0.001 | 1.44 | <0.001 | 1.47 | 1.25 - 1.74 | <0.001 |
| Pt age (per 10 yrs) | 1.17 | <0.001 | 1.19 | <0.001 | 18 | 1.10 - 1.26 | <0.001 |
| Pt race-ethnicity | cat | 0.08 | cat | 0.2 | w |  |  |
|  |  |  |  |  |  |  |  |
|  |  |  |  |  |  |  |  |
| **Crowns (Had only 1 follow-up visit)** |  |  |  |  |  |  |  |
| 20+ Patients | 3.47 | 0.02 | 3.58 | 0.02 | 3.58 | 1.77 - 7.23 | 0.02 |
| Pt female | 1.82 | 0.007 | 1.92 | 0.006 | 1.92 | 1.28 - 2.86 | 0.006 |
| Pt education (ordinal) | 1.31 | 0.06 | 1.33 | 0.045 | 1.33 | 1.03 - 1.73 | 0.045 |
|  |  |  |  |  |  |  |  |
|  | | |  |  |  |  |  |
| **Management of dentin hypersensitivity (MDH)** | | |  |  |  |  |  |
| Any FU |  |  |  |  |  |  |  |
| 10+ Patients | 2.12 | 0.03 | 2.11 | 0.03 | 2.11 | 1.20 - 3.72 | 0.03 |
| More than HS^6^ education | 2.21 | 0.04 | 2.31 | 0.03 | 2.31 | 1.26 - 4.24 | 0.03 |
| Midwest, South Atlantic, Northeast region | 2.78 | <0.001 | 2.74 | <0.001 | 2.74 | 1.55 - 4.85 | <0.001 |
|  |  |  |  |  |  |  |  |
| All FU |  |  |  |  |  |  |  |
| 10+ Patients | 1.57 | 0.03 | 1.65 | 0.01 | 1.7 | 1.21 - 2.38 | 0.009 |
| Pt age per 10 years | 1.09 | 0.07 | 1.08 | 0.1 | w |  |  |
| Pt: White, Asian vs other races | 1.62 | 0.002 | 1.6 | 0.004 | 1.61 | 1.22 - 2.14 | 0.004 |
| Midwest, Northeast | 2.51 | <0.001 | 2.48 | <0.001 | 2.51 | 1.84 - 3.42 | <0.001 |
|  |  |  |  |  |  |  |  |
|  | | | | |  |  |  |
| **Management of painful temporomandibular disorders (TMD)** | | | | |  |  |  |
| Any FU |  |  |  |  |  |  |  |
| Pt female | 1.87 | 0.09 | 1.89 | 0.09 | w |  |  |
| Pt age 55 or older | 2.51 | 0.003 | 2.51 | 0.003 | 2.51 | 1.22 - 5.17 | 0.003 |
|  |  |  |  |  |  |  |  |
| All FU |  |  |  |  |  |  |  |
| # of patients | cat | 0.046 | cat | 0.3 | w |  | X |
| Pt female | 1.76 | 0.005 | 1.74 | 0.006 | 1.78 | 1.27 - 2.48 | 0.004 |
| Pt age 55 or older | 1.56 | 0.005 | 1.53 | 0.008 | 1.511 | 1.08 - 2.10 | 0.009 |
| Bachelor’s degree or higher | 1.74 | <0.001 | 1.71 | <0.001 | 1.76 | 1.34 - 2.30 | <0.001 |
| Region | cat | 0.051 | cat | 0.3 | w |  |  |
|  |  |  |  |  |  |  |  |
|  | | | |  |  |  |  |

| **Supp Table 5 - continued** | | | |  |  |  |  |
| --- | --- | --- | --- | --- | --- | --- | --- |
|  | | | |  |  |  |  |
| **Predicting outcomes of root canal treatment (PREDICT)** | | | |  |  |  |  |
| Any FU |  |  |  |  |  |  |  |
| Dentist: White, Asian, Hispanic vs other | 4.00 | 0.005 | 2.14 | 0.01 | 2.08 | 1.42 - 3.05 | 0.01 |
| Owner non-solo or any PPO^7^ | 2.41 | 0.002 | 1.23 | 0.5 | w |  |  |
| Specialist | 2.50 | <0.001 | 1.64 | 0.06 | 1.65 | 1.09 - 2.49 | 0.025 |
| Pt female | 1.61 | 0.01 | 1.77 | 0.01 | 1.83 | 1.22 - 2.76 | 0.006 |
| Pt age (per 10 yrs) | 1.24 | 0.001 | 1.14 | 0.07 | 1.16 | 1.01-1.33 | 0.03 |
| Pt White/Asian | 2.43 | <0.001 | 1.65 | 0.02 | 1.74 | 1.18 - 2.58 | 0.009 |
| More than HS education | 2.24 | 0.001 | 1.57 | 0.05 | 1.67 | 1.15 - 2.41 | 0.02 |
| Midwest | 4.84 | <0.001 | 2.82 | 0.003 | 3.11 | 1.59 - 6.05 | <0.001 |
|  |  |  |  |  |  |  |  |
| All FU |  |  |  |  |  |  |  |
| Dentist: White, Asian, Hispanic vs other | 2.82 | 0.01 | 1.87 | 0.02 | 2.05 | 1.29 - 3.28 | 0.02 |
| PPO | 3.83 | 0.02 | 1.54 | 0.3 | w |  |  |
| Specialist | 1.70 | 0.006 | 1.37 | 0.04 | 1.35 | 1.04 - 1.75 | 0.04 |
| Pt Female | 1.59 | 0.001 | 1.76 | 0.002 | 1.72 | 1.29 - 2.30 | 0.001 |
| Pt age (per 10 yrs) | 1.12 | 0.01 | 1.09 | 0.09 | 1.12 | 1.02 - 1.23 | 0.02 |
| Pt White/Asian | 1.59 | 0.01 | 1.38 | 0.08 | w |  |  |
| More than HS education | 2.10 | <0.001 | 1.69 | 0.001 | 1.76 | 1.35 - 2.30 | <0.001 |
| Any dental insurance | 1.33 | 0.035 | 1.28 | 0.1 | w |  |  |
| Midwest | 3.78 | <0.001 | 2.33 | <0.001 | 2.69 | 1.98 - 3.65 | <0.001 |

^1^Individual: Adjusted only for patients clustered within practice using generalized estimating equations (GEE)

^2^Full model: Includes all individual characteristics with p<0.1

^3^Final reduced model: Backwards elimination (from full model) retaining only characteristics with p<0.05

^4^yrs:years

^5^Pt:Patient

^6^HS:High school,

^7^ PPO: Preferred provider organization (managed care)

^8^cat: categorical variable, no single odds ratio possible

^9^w:withdrawn because p>0.05
